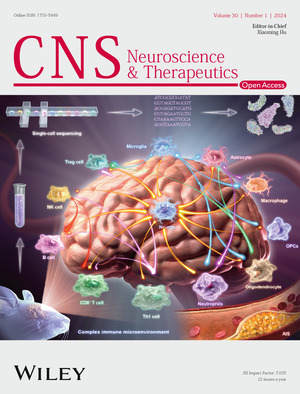# Additional Cover

**DOI:** 10.1111/cns.14623

**Published:** 2024-01-28

## Abstract

The cover image is based on the Review Article *Perspective from single‐cell sequencing: Is inflammation in acute ischemic stroke beneficial or detrimental?* by Xinpeng Deng et al., https://doi.org/10.1111/cns.14510.